# N-Methyl-D-Aspartate Receptor availability in First-Episode Psychosis: a multi-modal PET-MR brain imaging study

**DOI:** 10.1192/j.eurpsy.2022.238

**Published:** 2022-09-01

**Authors:** K. Beck, A. Arumuham, S. Brugger, R. Mccutcheon, M. Veronese, S. Kaar, T. Pillinger, J. Stone, O. Howes

**Affiliations:** 1 Institution of Psychiatry, Psychology & Neuroscience, Department Of Psychosis Studies, United Kingdom, United Kingdom; 2 Cardiff University Brain Research Imaging Centre (CUBRIC), School Of Psychology, HQ, United Kingdom; 3 Brighton and Sussex Medical School, University Of Sussex, Brighton, United Kingdom

**Keywords:** Psychosis, Neuroimaging, NMDAR, Glutamate

## Abstract

**Introduction:**

N-Methyl-D-Aspartate Receptor (NMDAR) hypofunction is hypothesised to underlie psychosis but this has not been tested early in illness.

**Objectives:**

Our aim was to determine if NMDAR availability was lower in patients with first episode psychosis compared to healthy controls.

**Methods:**

To address this, we studied 40 volunteers (21 patients with first episode psychosis and 19 matched healthy controls) using PET imaging with an NMDAR selective ligand, [^18^F]GE179, that binds to the ketamine binding site to index its distribution volume ratio (DVR) and volume of distribution (V_T_). Striatal glutamatergic indices (glutamate and Glx) were measured simultaneously using magnetic resonance spectroscopy imaging (^1^H-MRS).

**Results:**

Hippocampal DVR, but not V_T_, was significantly lower in patients relative to controls (p=0.02, Cohen’s d=0.81; p=0.15, Cohen’s d=0.49), and negatively associated with total (rho=-0.47, p= 0.04), depressive (rho=-0.67, p=0.002), and general symptom severity (rho=-0.74, p<0.001). Exploratory analyses found no significant differences in other brain regions (anterior cingulate cortex, thalamus, striatum and temporal cortex). We found an inverse relationship between hippocampal NMDAR availability and striatal glutamate levels in people with first-episode psychosis (rho = -0.74, p <0.001) but not in healthy controls (rho = -0.22, p = 0.44).

**Conclusions:**

These findings are consistent with the NMDAR hypofunction hypothesis and identify the hippocampus as a key locus for relative NMDAR hypofunction, although further studies should test specificity and causality.

**Disclosure:**

No significant relationships.

